# Thermal, Mechanical, and Acoustic Properties of Polydimethylsiloxane Filled with Hollow Glass Microspheres

**DOI:** 10.3390/ma15051652

**Published:** 2022-02-23

**Authors:** Sergei Vlassov, Sven Oras, Martin Timusk, Veronika Zadin, Tauno Tiirats, Ilya M. Sosnin, Rünno Lõhmus, Artis Linarts, Andreas Kyritsakis, Leonid M. Dorogin

**Affiliations:** 1 Institute of Physics, University of Tartu, W. Ostwaldi Str. 1, 50412 Tartu, Estonia; martin.timusk@ut.ee (M.T.); rynno.lohmus@ut.ee (R.L.); 2 Tartu College, Tallinn University of Technology, Puiestee 78, 51008 Tartu, Estonia; sven.oras@ut.ee; 3 Institute of Technology, University of Tartu, Nooruse 1, 50411 Tartu, Estonia; veronika.zadin@ut.ee (V.Z.); tauno.tiirats@ut.ee (T.T.); andreas.kyritsakis@ut.ee (A.K.); 4 Research Laboratory of Functional Materials Technologies, Faculty of Materials Science and Applied Chemistry, Riga Technical University, Paula Valdena 3/7, 1048 Riga, Latvia; 5 Institute of Advanced Systems for Data Transmission, ITMO University, Kronverskiy pr., 49, 197101 Saint-Petersburg, Russia; sim.nanosci@gmail.com (I.M.S.); lmdorogin@itmo.ru (L.M.D.); 6 Institute of Technical Physics, Faculty of Materials Science and Applied Chemistry, Riga Technical University, Paula Valdena 3/7, 1048 Riga, Latvia; artis.linarts@rtu.lv

**Keywords:** PDMS, hollow glass microspheres, thermal conductivity, sound insulation

## Abstract

Polydimethylsiloxane (PDMS) is the most widely used silicon-based polymer due to its versatility and its various attractive properties. The fabrication of PDMS involves liquid phase cross-linking to obtain hydrophobic and mechanically flexible material in the final solid form. This allows to add various fillers to affect the properties of the resulting material. PDMS has a relatively low Thermal Conductivity (TC), in the order of 0.2 W/mK, which makes it attractive for thermal insulation applications such as sealing in construction. Although a further decrease in the TC of PDMS can be highly beneficial for such applications, most research on the thermal properties of PDMS composites have focused on fillers that increase the TC rather than decrease it. In the present work, we propose a simple and reliable method for making a PDMS-based composite material with significantly improved thermal insulation properties, by adding hollow glass microspheres (HGMs) to the mixture of the liquid base and the cross-linker (10:1 ratio), followed by degassing and heat-assisted crosslinking. We obtained a 31% reduction of thermal conductivity and a 60% increase in the elastic modulus of samples with HGM content of 17% by weight. At the same time, the sound insulation capacity of the PDMS-HGM composite is slightly decreased in comparison to pure PDMS, as a result of its lower density. Finally, the wettability of the samples had no dependence on HGM content.

## 1. Introduction

Polydimethylsiloxane (PDMS) is the most widely used silicon-based polymer due to its versatility and the whole range of attractive properties that include, but are not limited to, transparency [1], mechanical flexibility [2], relatively high chemical and thermal stability [3], low toxicity, low solubility in water, and high dielectric constant. Furthermore, the nonpolar (hydrophobic) surface of PDMS protects photocatalysts from bio-fouling [4]. The energy of Si-O bond is 443 kJ/mol [5], so PDMS is stable under solar irradiation. 

Fabrication of PDMS starts from the liquid phase followed by cross-linking to obtain final solid material. This gives opportunity to tune the properties of resulting composite relatively easily by adding other liquids and/or powders into a liquid precursor before cross-linking. One of the apparent properties that can be easily modified by fillers in both directions is thermal conductivity (TC) [6,7]. In certain applications of PDMS, enhancement of TC is needed, for instance to improve heat dissipation when PDMS is used as a sealant in electronics. Carbon-based fillers are considered as the most promising filler candidate [8,9,10,11]. Addition of 4.2 wt% of aligned graphene tubes results in 1082.14% higher TC compared to pure PDMS [7]. One of the most studied enhancer of TC used in PDMS is carbon nanotubes (CNTs) [12,13,14,15]. Other fillers reported in literature include carbon black (CB) [16,17], carbon black and CNTs mixture [18], metallic powder [19] and metallic nanowires [20]. 

Fillers can also increase thermal stability of PDMS at elevated temperatures. Studied filler materials include nanoclay [21], nano-silica [22,23], zirconia or zirconium carbide [24] and others. However, not much works can be found on decreasing TC of PDMS possibly due to already relatively low thermal conductivity of pure PDMS (in the order of 0.2 W/K [25,26]). Nevertheless, further decrease in TC of PDMS can be beneficial in applications where thermal insulation is required like e.g., sealing in construction. 

One of the most promising filler materials for thermal insulation in general is silica aerogel [27,28,29,30,31] due to a low density (0.003–0.1 g/cm^3^), high porosity (>99%), and low thermal conductivity (0.013–0.04 W/mK). To prevent the absorption of liquid from matrix material during the composite fabrication stage, aerogel should be made either strongly hydrophobic or hydrophilic (depending on matrix composition) e.g., by chemical treatment or additional fabrication steps should be introduced to preserve pores during mixing and solidifying [29,30,31]. Otherwise, addition of aerogel could even lead to increased TC of the final product [25,32]. Recently, excellent results in reducing the TC of PDMS-silica aerogel composite were obtained by Lee et al. [32] by filling of aerogel pores with ethanol before mixing with PDMS to prevent pores of the aerogel from becoming impregnated with PDMS matrix. After curing at 80 °C, the ethanol was removed from composite by evaporation and diffusion. Authors succeeded to decrease the heat conductivity of their composite down to 0.018 W/mK.

Despite the high efficiency of silica aerogel fillers, introduction of additional steps makes process more complicated that is not always acceptable for “on-site” fabrication in non-laboratory conditions. Moreover, silica aerogel has a relatively high price due to the need for sophisticated equipment and the long drying process with supercritical fluids [31]. Fragile behavior under relatively low external forces is another limiting factor in the use of silica aerogels. Low mechanical robustness is the problem relevant also for various porous forms of PDMS. It was shown that for porosity in the range of approx. 70–80% compressive modulus decreases 2 orders of magnitude compared to solid PDMS (from few MPa to few tens of kPa) [33].

Another candidate as filler for thermally insulating composites is hollow glass microspheres (HGMs) [34,35,36]. Similarly to silica aerogels, HGMs have a low density and excellent thermal insulation properties. However, contrary to silica aerogels, HGMs exhibit a high specific compressive strength and stiffness due to the spherical shape [37]. Moreover, HGMs is more cost-efficient material due to significantly lower manufacture costs compared to aerogels. Encapsulation of gas inside closed rigid spheres prevents them from filling with the matrix material [25]. However, special care still should be taken to avoid breaking of HGMs, since it may result in increase of TC up to values exceeding that of a pure matrix material without filler as was shown by Hu et al. [38] for silicon rubber (vulcanized product of PDMS) filled with HGMs (diameter 10–100 µm). 

In the present work, we describe fabrication and characterization of composite materials based on PDMS filled with HGMs at various concentrations. Reported composite material benefits from simplicity of preparation and offers good heat insulating properties without significantly sacrificing mechanical robustness and sound insulating properties. Such characteristics make it attractive for applications like e.g., sealing in construction.

## 2. Materials and Methods

### 2.1. Preparation of Composites

PDMS matrix was made from Sylgard 184 Silicone Elastomer Kit (Dow Corning, Midland, MI, USA), consisting of two liquid components: the base and the curing agent. According to Gonzales et al. [39] the curing agent consist of dimethylmethylhydrogen siloxane dissolved in vinyl end-terminated poly(dimethylsiloxane). Hollow glass microspheres (HGMs) (Q-CEL 300, Potters) with tamped bulk density of 0.12 g/cm^3^ and particle size ranging from 5 to 200 µm (90 µm in average) were used as a heat insulating filler for PDMS matrix. PDMS composites were prepared by adding a predetermined amount of HGMs to PDMS into a freshly prepared liquid mixture of base and curing. Mixing was performed manually with plastic rod to avoid excessive damaging of HGMs by mixing procedure. Base to curing agent ratio was kept at 10:1 for all samples as the most common ratio in PDMS applications. Freshly prepared mixture of base, current agent and HGMs was degassed at 300 mbar until no air bubbles remained. Degassed mixture was then poured into the specially prepared vertical cell consisting of two 8 × 8 cm^2^ glass plates separated by 3.04 mm thick Teflon spacers and sealed with 5 mm silicone gasket so that the cell is tightly closed from 3 sides and is opened from above. Glass plates were pressed together and connected by metal clamps. Glass cells filled with PDMS-HGMs mixture were placed into the laboratory furnace for 1 h at 70 °C. Several samples were prepared with varying concentration of HGMs in PDMS, 0%, 2.5%, 5%, 10% and 17% by weight with respect to PMDS. 

### 2.2. Characterization

The distribution of HGMs inside solid PDMS was studied with scanning electron microscope (SEM) (VEGA II, Tescan, Brno, Czech Republic). Samples for SEM imaging were prepared by tearing small pieces of the material from bigger samples to create fresh surfaces for analysis while avoiding damaging the HGM’s that would occur in case of cutting by blade. 

Density of the samples was determined by measuring the mass and volume of the samples. All samples were 3.04 mm in thickness and 12.9 mm in diameter for density measurements.

Thermal conductivity was measured according to ASTM 1114-98 standard by using commercial device THASYS by Hukseflux Thermal Sensors B.V. (Delft, the Netherlands).

Acoustic measurements were performed at 1 kHz sine wave with dynamic microphone Rode M1-S. Sound source was placed in a special soundproof box that has opening 4 cm in diameter. The opening was covered with one of the samples and reduction in sound level compared to uncovered opening was registered. Thickness of the samples was 5.08 mm.

Wettability of the samples was tested by drop-casting three 100 µL droplets on each sample and measuring the average contact angle.

Mechanical properties of the samples were measured with Zwick/Roell Z2.5 (Ulm, Germany) materials testing machine in compression tests performed according to ASTM d575 standard: measurements were performed at ambient temperature; each sample was tested 3 times at compression rate 10 mm/min and maximum compression force 750 N; calculation of *E* modulus (linear fit of stress vs. strain graphs) was done only from the 3rd test. Samples had a cylindrical shape 12.5 mm in height and 28.6 mm in diameter. Exact thickness of the samples was measured by the test equipment under compression force of 3 N.

### 2.3. Simulations

Finite element method (FEM) simulations of the composite were conducted assuming heat equation in steady state. All materials were modelled directly as geometric primitives: glass microspheres were inserted with their geometrical parameters (diameter, wall thickness) adopted from experimental data. Glass spheres were assumed to be filled with air and were arbitrarily placed into PDMS matrix. Sample was simulated as cube shaped PDMS block with unit edge size. For the heat conductivity calculations constant temperature boundary conditions were used at the left and right side of the PDMS block, all other boundaries were considered thermally insulating, replicating, thus modelling symmetry boundary conditions and replicating the block in all directions. Temperature difference between constant temperature boundaries was 10 °C with 20 °C at lower limit. As a result of simulation, average heat flux at boundaries was calculated followed by the application of Fourier’s law of heat conduction to evaluate the homogenized heat transfer coefficient of the composite. For the simulation of the compression test, the momentum equation was solved in a unit domain, having three coordinate planes defined as periodic symmetry planes (to simulate a larger domain). Linearly varying displacement was defined on the top surface. The Young’s modulus was evaluated as the slope of the resulting force-displacement curve. Simulations were performed in linear elastic regime and were verified with nonlinear (Neo-Hookean) PDMS material. It is assumed that spheres will break before non-linear effects become significant. The inserted spheres were considered fully bonded with the PDMS material and in average 8 spheres per unit domain were considered. A linear tetrahedral mesh with locally refined elements was used. Based on a mesh convergence study, 70,000 volumetric elements were used. All calculations were conducted using Comsol Multiphysics 5.5.

## 3. Results and Discussion

**Figure 1:**
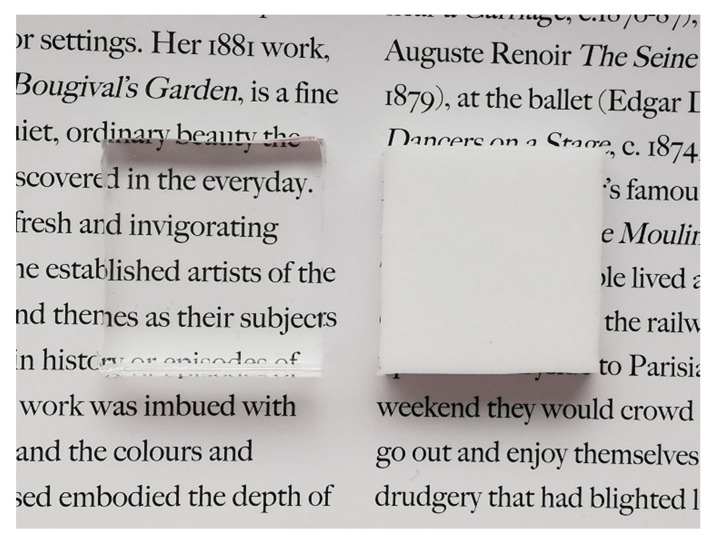
Pure PDMS (**left**) and PDMS with 17 wt% HGMs (**right**). Thickness of both samples is 5.8 mm.

Addition of HGMs gave the PDMS a white coloration already at 2.5% concentration due to intense light scattering by the composite, arising from the mismatch of refractive indices of PDMS matrix and HGM’s. At 17% HGM’s content 5.8 mm thick samples became completely opaque (Figure 1). 

Surface wettability can be important characteristic in sealing applications, as hydrophobic sealants are less likely to leak in case of minor damage. Wettability can be estimated by measuring the water droplet contact angle. Measurements performed on our composites did not show any considerable dependence of surface wettability on HGM’s content (see Table 1) thus retaining hydrophobic characteristic of pristine PDMS. This is logically explained by the fact that HGMs are entirely embedded into PDMS matrix, so the surface of the composite is basically a pure PDMS.

**Figure 2:**
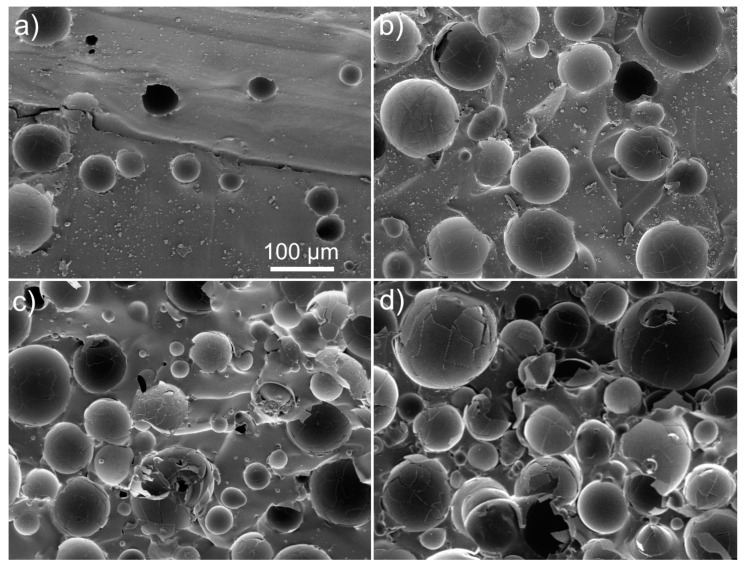
SEM micrographs of PDMS filled with HGMs: 2.5% (**a**), 5% (**b**), 10% (**c**), 17% (**d**).

**Figure 3:**
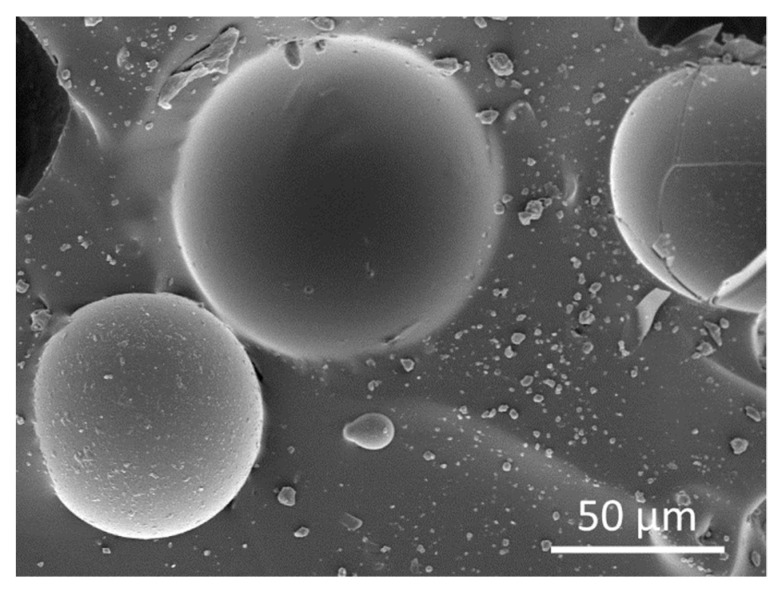
SEM micrograph of HGMs embedded in PDMS. The surface for the observation was obtained by tearing the cured PDMS containing 5% of HGMs.

SEM observations of torn-off PDMS-HGMs slices revealed that a large fraction of HGMs are broken (Figure 2). It is difficult to say if fracturing happened during the mixing and heating stages, or as a result of the sample preparation for SEM imaging. Although the slices with fresh surfaces for SEM analysis were obtained by tearing, not cutting, the process still involves significant mechanical stresses. Most probably, both mixing and tearing were responsible for damage of HGMs. However, contribution of tearing seems to be more significant as closer look reveals that most HGMs that are embedded deeper into the surface of PDMS look undamaged in SEM images (Figure 3). 

Broken spheres give opportunity to measure the wall thickness. However, if HGMs have different wall thicknesses then the thin-walled spheres are expected to break easily and therefore will prevail in SEM images and alter the estimation of average wall thickness. For a more realistic estimation of wall thicknesses, we intentionally mechanically damaged pure HGMs and imaged them in SEM (Figure S1 in SI). Median average wall thickness calculated from 54 broken fragments is 766 µm. The size of the microspheres also seems to be an important factor in their structural integrity: the majority of sub-100 µm spheres appeared intact under SEM observation (Figure 3).

**Figure 4:**
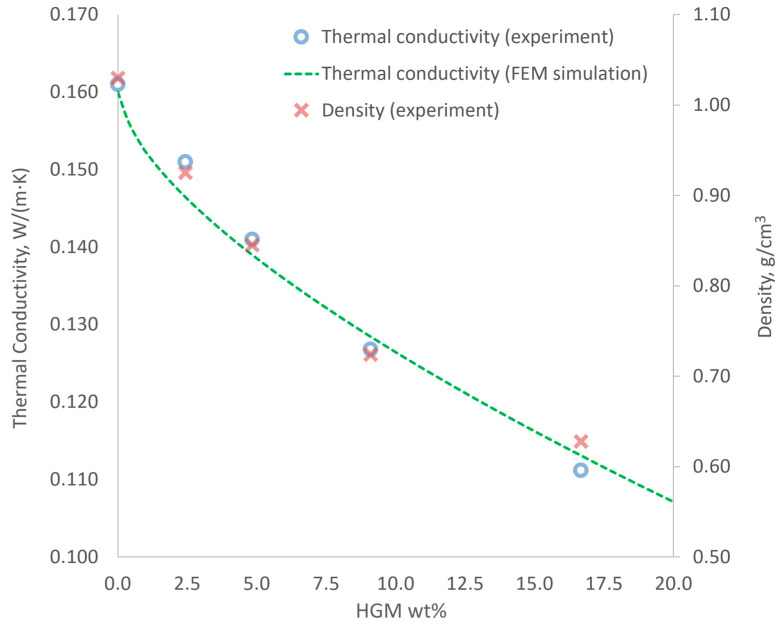
The dependence of thermal conductivity and density of PDMS-HGMs composite on HGMs content.

The dependence of TC and density of PDMS-HGMs composite on HGMs content, alongside with results of FEM simulation, are shown in Figure 4. It can be seen that both the TC and density are steadily decreasing with increasing HGM content, in a good agreement with FEM results. These facts suggest that significant amount of HGMs inside PDMS remained hollow; otherwise, we would observe increase in both TC and density caused by solid glass shatters that are heavier and better heat conduct than PDMS. In total, we achieved approx. 31% reduction in TC (from 0.161 to 0.111 W/(m⋅K)) at 17 wt% HGMs content. This result is far from the one obtained by Lee et al. [32] for specially treated aerogel filler. However, the use of HGMs benefits from simplicity of fabrication process as it does not require additional steps for preserving hollow structure and therefore can be performed outside of the laboratory conditions without the use of special equipment. In contrast, addition of silica aerogel without the pore restoration procedure results in increased TC of PDMS and other organic materials [25,32]. Therefore, the use of HGMs instead of silica aerogel particles can be justified in certain practical applications such as on-site preparation of sealer with enhanced thermal insulation. It should be noted that addition of HGMs above 17 wt% is problematic due to high viscosity of the solution making it difficult to pour. 

Porosity was not directly measured in the present study, however considering that HGMs have low density even in a tamped form (0.12 g/cm^3^), and median wall thickness of the spheres is just in the order of 1% of the sphere diameter, in first approximation we can estimate porosity from the density of the samples. Thus, at 17 wt% HGM content the estimated porosity in approx. 30%. 

**Figure 5:**
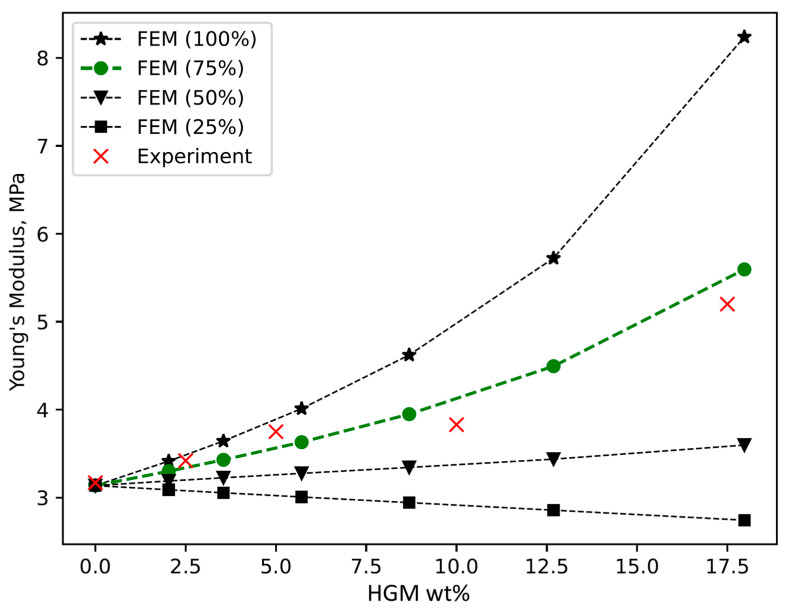
The dependence of the Young’s modulus of PDMS-HGMs composite on HGMs content. Experimental results (red crosses) are given alongside with FEM calculations.

Next, the Young’s modulus of the samples was experimentally measured in compression test. It was found that at maximum HGM content (17%), the stiffness of the sample increased approximately 1.6 times. Results are presented as red crosses on the graph in Figure 5. Additionally, test conditions were simulated by FEM revealing that if we assume 100% of spheres survive mixing and compression test then expected stiffness of the samples filled with HGMs should significantly exceed the value found in experiment. From the TC and density data, we know that most of the spheres survive the mixing and polymerization procedure. Therefore, we suppose that some considerable number of spheres are broken during the compression test. Ideally, HGMs should greatly increase the stiffness of PDMS, since Young’s modulus of silicon glass is four orders of magnitude higher than that of a PDMS [40]. However, if spheres are broken and lose their structural integrity in already solidified sample, then they can be considered as spherical voids that have negative effect on elastic modulus. Therefore, FEM simulations can be used as a tool for estimation of the percentage of broken HGMs in the composite. Good agreement between measured and simulated results were found when the content of undamaged spheres in PDMS in FEM model was around 75% of all added spheres. Moreover, by extrapolating the magnitude of material damage, we also see that if only 25% of the spheres remain intact, then Young’s modulus of the composite will be even lower than that of a pure PDMS as it effectively turns PDMS into porous material. 

Here we would like to briefly discuss some details and potential limitations of our FEM simulations. In our model, spheres were positioned arbitrary. However, due to the localized nature of the geometrical set-up, the simulations demonstrate considerable sensitivity to the placement of the spheres, potentially making it possible to reveal additional correlations and trends if more accurate analysis is needed. Moreover, we used average sphere size obtained from SEM observations, while in reality diameters are highly scattered. Finally, to obtain matching weight ratios between experiment and simulation, wall thicknesses used in the model had to be 6 times higher than the average wall thickness found from SEM observation of spheres broken during mixing and sample preparation stage for SEM imaging. This inconsistency becomes logical if we consider the fact that in the SEM observations, we could measure the wall thickness only of the broken spheres, and it is reasonable to assume that the thinner is the wall the higher is the probability of damaging the sphere during mixing and SEM sample preparation. Therefore, wall thicknesses measured in SEM are most probably strongly biased towards the lower values. Similar logic should be applied also to the compression test—supposedly thin-walled spheres are broken during the measurements and the resultant stiffness of the sample is defined by the interplay between voids left from broken thin-walled spheres and undamaged thick-walled spheres. This leads us to the scenario similar to the one we have in our FEM simulations, i.e., that actually remaining spheres have significantly thicker walls than those we see in broken spheres in SEM images. From here, we can conclude that by implementing more rigorous control of preparation of HGMs by selectively removing too thin spheres, both improvement of thermal and elastic properties could potentially be reached. 

If we now try to estimate the influence of sphere breaking on TC of the composite, then even if consider extreme scenario where all spheres will be damaged during the exploitation of the material, no considerable worsening of its thermal insulating properties is expected, as PDMS is already solidified, so there will be microscopic voids in place of glass spheres. Moreover, TC may potentially decrease further, if spheres lose their structural integrity, as it creates additional resistance for the heat transfer along the glass surface. However, additional studies are needed to evaluate this effect quantitatively. 

Finally, sound insulation of the samples—another relevant property in sealing applications—was measured. Our measurements have shown that addition of HGMs resulted in slightly decreased sound insulation capacity from −29 dB for pure PDMS to −26 dB for PDMS with 17 wt% HGMs as tested on 5.08 mm thick samples for 1 kHz sound frequency (commonly used frequency in acoustic tests as it corresponds well to the frequency of human voice). The ability to absorb sound energy is related both to structure and to density (mass) of the object. In our case, higher density of pure PDMS was determining factor for more efficient sound insulation.

## 4. Conclusions

A novel composite material with low thermal conductivity have been prepared by adding hollow glass microspheres (HGMs) to the mixture of liquid base and cross-linker (10:1 ratio) of PDMS. HGMs concentration in the samples was varied from 0% to 17% by weight. Addition of HGM’s gave PDMS an opaque white appearance. Water droplet contact angle measurements did not show any considerable dependence of surface wettability on HGMs content retaining the hydrophobic properties of pristine PDMS. Thermal conductivity and density steadily decreased with increase of HGM’s concentration, reaching 31% reduction in thermal conductivity (from 0.161 to 0.111 W/(m⋅K)) and 39% reduction in density (form 1.03 to 0.63 g/cm^3^) at 17% HGM’s concentration in comparison to pure PDMS, while Young’s modulus increased approx. 60%. Sound insulation capacity of 5.08 mm thick samples decreased from −29 dB for pure PDMS to −26 dB for PDMS with 17% HGMs content as measured at 1 kHz sound frequency. Thermal conductivity values obtained in this work were higher as compared to those obtained by Lee et al. [32] for specially treated aerogel filler. However, the use of HGM’s in preparation of composites with PDMS benefits from simplicity of fabrication as it does not require additional steps for preserving hollow structure and therefore can be performed outside of the laboratory conditions without the use of special equipment. Therefore, the use of HGMs instead of silica aerogel particles can be justified in certain practical applications such as on-site preparation of sealer with enhanced thermal insulation. 

## Figures and Tables

**Table 1:** Results of the water droplet contact angle measurements.

**HGMs Content, %**	**0**	**2.5**	**5**	**10**	**17**
cont. angle 1	112	116	113	118	114
cont. angle 2	116	118	119	118	116
cont. angle 3	114	117	116	115	118
**Average angle**	**114 ± 2**	**117 ± 1**	**116 ± 3**	**117 ± 2**	**116 ± 2**

## Data Availability

The data presented in this study are available on request from the corresponding author.

## Supplementary Materials

The following supporting information can be downloaded at: https://www.mdpi.com/article/10.3390/ma15051652/s1, Figure S1: SEM micrographs of broken HGMs.
