# Supplementary material for: Thermal, Mechanical, and Acoustic Properties of Polydimethylsiloxane Filled with Hollow Glass Microspheres
Source: Materials (Basel). 2022 Feb 23;15(5):1652. doi: 10.3390/ma15051652 (PMC8910890; doi:10.3390/ma15051652)
Supplement: Supplementary file 1 [file materials-15-01652-s001.zip › materials-1557786-SI.pdf]

Thermal, Mechanical, and Acoustic Properties of Polydimethylsiloxane Filled with Hollow Glass Microspheres

Sergei Vlassov [1,*], Sven Oras [2,3], Martin Timusk [1,4], Veronika Zadin [3], Tauno Tiirats [3], Ilya M. Sosnin [5], Rünno Lõhmus [1], Artis Linarts [6], Andreas Kyritsakis [3], Leonid M. Dorogin [5]

1 Institute of Physics, University of Tartu, W. Ostwaldi Str. 1, 50412, Tartu, Estonia
2 Tallinn University of Technology, Tartu College, Puiestee 78, Tartu, 51008, Estonia
3 Institute of Technology, University of Tartu, Nooruse 1, 50411, Tartu, Estonia
4 Research Laboratory of Functional Materials Technologies, Faculty of Materials Science and Applied Chemistry, Riga Technical University, Paula Valdena 3/7, 1048 Riga, Latvia
5 Institute of Advanced Systems for Data Transmission, ITMO University, Kronverskiy pr., 49, 197101 Saint-Petersburg, Russia
6 Institute of Technical Physics, Faculty of Materials Science and Applied Chemistry, Riga Technical University, Paula Valdena 3/7, Riga, 1048, Latvia
* Corresponding: sergei.vlassov@ut.ee (S. Vlassov) Tel.: +37255941841

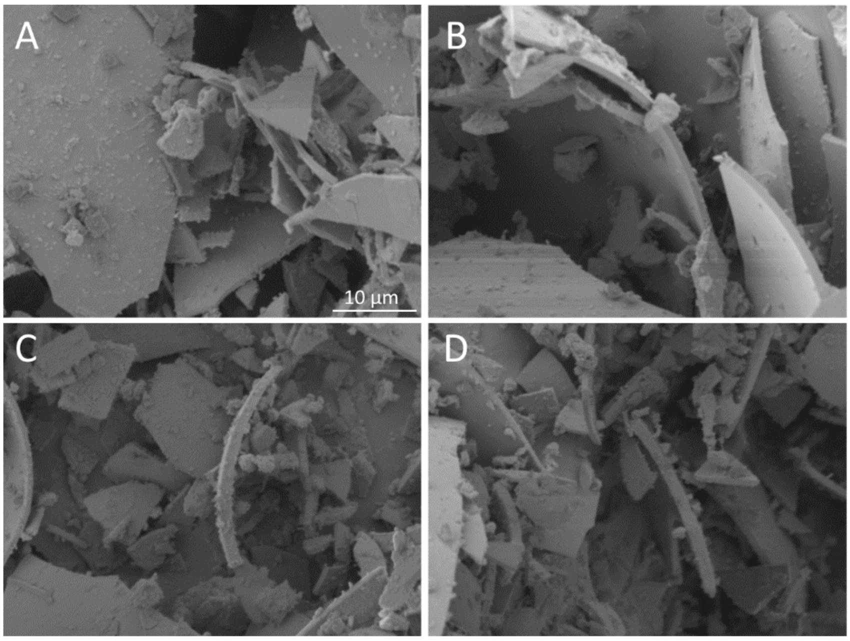

Figure S1. SEM micrographs of broken HGMs. A, B, C and D are images taken in deferent places of the same sample.
